# On a Model of Rumors Spreading Through Social Media

**DOI:** 10.3390/e27090903

**Published:** 2025-08-26

**Authors:** Laurance Fakih, Andrei Halanay, Florin Avram

**Affiliations:** 1 Department of Mathematics and Informatics, University Politehnica of Bucharest, 060042 Bucharest, Romania; andrei.halanay@upb.ro; 2 Laboratoire de Mathématiques Appliquées, Université de Pau, 64012 Pau, France; florin.avram@univ-pau.fr

**Keywords:** rumor dynamics, mathematical modeling, delay differential equations, online social media, misinformation, multistrain rumor model, stability analysis

## Abstract

Rumors have become a serious issue in today’s modern era, particularly in view of increased activity in social and online platforms. False information can go viral almost instantaneously through social networks, which immediately affect society and people’s minds. The form of rumor it develops within, whether fabricated intentionally or not, impacts public perspectives through manipulation of emotion and cognition. We propose and analyze a mathematical model describing how rumors can spread through an online social media (OSM) platform. Our model focuses on two coexisting rumors (two strains). The results provide some conditions under which rumors die out or become persistent, and they show the influence of delays, skepticism levels, and incidence rates on the dynamics of information spread. We combine analytical tools (Routh–Hurwitz tests and delay-induced stability switches) with MATLAB/Python simulations to validate the theoretical predictions.

## 1. Introduction

Rumors have always been a fundamental aspect of human communication for centuries, having a deep impact on perception, public opinion, and frequently playing an important role in social and political life. However, with improvements in digital technology and social media platforms, the speed at which rumors spread has reached unprecedented levels. The explosion of social networks has changed the way information is shared, making it possible for real and false information to reach large groups of people. This development raises serious concerns about the accuracy of online content and the potential dangers of misinformation.

Rumors can come from a variety of sources, including false information, unverified press releases, and individuals seeking attention.

While not all of them cause immediate harm, many have the potential to cause panic, distort public perception, and destroy one’s reputation. There is a critical necessity to investigate how rumors spread in order to develop effective strategies for fighting disinformation and creating an informed society.

Rumor spreading in social networks and communities has long been a subject of interest in both sociology and mathematics. Understanding how information, whether true or false, spreads within a population can help to devise some strategies to control misinformation and improve communication channels. Mathematical models have proved to be an important tool in describing and predicting rumor dynamics. One of the earliest models, introduced by Daley and Kendall in [1], classifies the population into three categories: ignorants (those who have not heard the rumor), spreaders (those who actively spread it), and stiflers (those who know the rumor but no longer spread it).

Based on this work, researchers have introduced more complex models by including various realistic factors. Time delays, for example, represent the time a person takes to change states, such as from hearing a rumor to spreading or becoming skeptical. Such delays have a huge impact on the stability of the system and the final result of the rumor propagation process [2]. Other important concerns are the ways to include control strategies into rumor models, as timely interventions can limit the spread of harmful misinformation [3].

In this work, we extend the current models by considering several types of rumors that are spreading at the same time, each with its own skepticism and recovery rates. We introduce an eight-variable delay differential system representing different states of individuals and analyze the stability of its equilibria for various conditions of parameters. Our goal is to understand the propagation of multistrain rumors and the variables that affect stability.

### 1.1. Modeling Assumptions and Mean-Field Viewpoint

In plain terms, our model looks at the platform as a whole and asks how the shares of people in each group change over time. We do not track who talks to whom; instead, we assume everyone mixes randomly (a standard “mean-field” view; see [4]). This choice keeps the math transparent and lets us state clear threshold conditions, at the cost of ignoring detailed network features (friend graphs, communities, or highly connected hubs).

We rely on a few simple, realistic assumptions:

- We track fractions, not individuals: Each variable measures the proportion of users in a state (active, skeptical, believer of rumor 1/2, etc.).
- Contacts saturate: People have limited time and attention, so the effective contact rate does not grow without bound. This is why our incidence terms are “saturating.”
- Skepticism is a temporary stop: The skeptic group acts like a short holding area: users who stop believing a rumor may spend some time skeptical before disconnecting or moving on.
- One dominant delay: We model a single characteristic delay τ that captures the leading feedback between active users and later disengagement. This is the dominant timescale we observe in our scenarios. Heterogeneity in response times can be handled with a distributed kernel and is discussed explicitly below; the main stability picture (first Hopf crossing and subsequent windows) remains the same for small dispersion.

This simplified picture is widely used as a first step: it gives interpretable formulas and stable/unstable regimes that are easy to test. More detailed, network-based models can be layered on top later if one wants to add communities, degree heterogeneity, or multiple overlapping platforms.

### 1.2. Related Work and Contributions

In brief, there is already work on the simultaneous spread of multiple rumors [5,6,7], how delays affect rumor dynamics and how they can be controlled [8,9,10,11], and the interaction between rumors and anti-rumors [12]. For broader overviews, see the surveys in [13,14,15].

#### What Is New Here

Our paper adds the following:

- **A compact two-rumor model with three realistic features:** We combine (i) *saturating incidence* (sharing cannot grow without bound because attention/time are limited), (ii) a *skepticism reservoir* that can feed disconnection, and (iii) a *single dominant delay* for the active disconnected loop. This specific combination, in this simple form, is not present together in the cited studies.
- **Clear conditions for rumor persistence:** We show that the endemic state (rumors present) reduces to a *single quadratic in* $x_{2}^{*}$, from which we read off when the rumor persists and a *positivity condition* for the disconnected class $x_{3}^{*}$. These are easy to check and interpret.
- **Straightforward stability analysis:** Using a *rank-one perturbation* argument, the 8D characteristic equation splits into a 3×3 *delayed core* plus a rumor block. This lets us apply standard tests: Routh–Hurwitz at τ=0 and classical *stability-switch* criteria for τ>0 (Cooke–van den Driessche; Beretta–Kuang) with transparent algebra.
- **Delay-driven oscillatory windows:** We compute the first critical delay (τ≈1.49) where a *Hopf bifurcation* appears, and we numerically show that stability can be lost and then regained as τ grows (multiple switches). We also explain the mechanism: a *delayed negative feedback* in the active–disconnected loop; with two rumor components, a second oscillatory mode can modulate amplitudes.

This mean-field framework does not replace network-explicit models; it *complements* them by giving readable formulas, interpretable thresholds, and quick “what-if” scenarios that can be extended later to networks with degree heterogeneity, communities, or overlapping platforms.

## 2. Two Rumors Spreading in an OSM

In this model, we assume there are two rumors (Rumor 1 and Rumor 2) spreading among the users of a social media platform. We track the following eight state variables:

- $x_{1}$: **Potential users of OSM** (individuals who could join but have not yet joined);
- $x_{2}$: **Active users of OSM** (currently using the platform, susceptible to rumors);
- $x_{3}$: **Non-users (disconnected)** from OSM who have abandoned and never joined the platform again, after a delay;
- $x_{4}$: **Considering Rumor 1** (they heard Rumor 1 and are thinking about it);
- $x_{5}$: **Considering Rumor 2**;
- $x_{6}$: **Believers of Rumor 1** (those actively spreading or convinced by rumor 1);
- $x_{7}$: **Believers of Rumor 2**;
- $x_{8}$: **Skeptics for either rumor** (individuals who do not believe either rumor).

The system of equations is as follows:

$$\;\;\;\;\;\;\;\;\;\;\left\{\begin{array}{llllllllllllllllll} {\dot{x}}_{1}=\Lambda -\alpha x_{1}x_{2}-\mu x_{1}, & & & & & & & & & & & & & & & & & (1)\\ {\dot{x}}_{2}=\alpha x_{1}x_{2}-\eta x_{2}\;x_{3}\left(t-\tau \right)-\mu x_{2}, & & & & & & & & & & & & & & & & & (2)\\ {\dot{x}}_{3}=\eta x_{2}\;x_{3}\left(t-\tau \right)-\mu x_{3}+\omega x_{8}, & & & & & & & & & & & & & & & & & (3)\\ {\dot{x}}_{4}=\frac{\beta_{1}x_{2}x_{6}}{1+\alpha_{1}x_{2}+\epsilon_{1}x_{6}}-\gamma_{1}x_{4}, & & & & & & & & & & & & & & & & & (4)\\ {\dot{x}}_{5}=\frac{\beta_{2}x_{2}x_{7}}{1+\alpha_{2}x_{2}+\epsilon_{2}x_{7}}-\gamma_{2}x_{5}, & & & & & & & & & & & & & & & & & (5)\\ {\dot{x}}_{6}=\gamma_{1}x_{4}-\mu_{1}x_{6}, & & & & & & & & & & & & & & & & & (6)\\ {\dot{x}}_{7}=\gamma_{2}x_{5}-\mu_{2}x_{7}, & & & & & & & & & & & & & & & & & (7)\\ {\dot{x}}_{8}=\mu_{1}x_{6}+\mu_{2}x_{7}-\omega x_{8}. & & & & & & & & & & & & & & & & & (8) \end{array}\right.$$

All parameters are non-negative, and τ>0 is a delay. We write the delay explicitly as $x_{3}\left(t-\tau \right)$ throughout. Our goals are to prove first that the solutions are essentially positive and then to identify the equilibria of this system and study their stability by looking at the linear approximation.

### 2.1. Delay Structure: Single Versus Distributed Delays

We use a *single* characteristic delay τ to represent the typical time between a rise in activity and the subsequent increase in disengagement. This choice keeps the analysis explicit (closed criteria at τ=0 and standard stability–switch tests when τ>0) and isolates the dominant feedback that triggers oscillations.

If response times are heterogeneous, the same feedback can be written with a distributed delay

$$\int_{0}^{\infty }K\left(\theta \right)\;x_{3}\left(t-\theta \right)\;d\theta ,$$

with *K* a probability kernel (e.g., Erlang/Gamma). Linearizing gives a characteristic equation of the form

$$P\left(\lambda \right)+Q\left(\lambda \right)\;\hat{K}\left(\lambda \right)=0,$$

so the usual geometric stability-switch criteria still apply (Cooke–van den Driessche; Beretta–Kuang). In tests with moderate dispersion (Erlang/Gamma *K*), the transition is smoothed, but the qualitative picture is unchanged: the same first Hopf threshold and the same sequence of stability windows as in the single-delay case. For strongly dispersed *K*, thresholds may shift, but the delay-driven mechanism remains the driver of oscillations.

### 2.2. On Cross-Rumor Interactions and Model Scope

In principle, the two rumors may interact through (i) *competition*/*promotion* (exposure to one rumor decreases/increases the propensity to consider the other) and (ii) *switching* between believer groups. We intentionally analyze a *minimal* two-rumor core *without* explicit cross-promotion/competition. This parsimony yields closed-form endemicity conditions, preserves a block structure that enables the rank-one perturbation analysis, and avoids introducing several hard-to-identify parameters.

If cross-effects are required, they can be incorporated in standard ways (cf. [7,12]):

- *Competition/promotion in consideration* $\left(x_{4},x_{5}\right)$: multiply the existing saturating incidence by a bounded modifier, e.g., $\frac{1+\rho_{21}x_{7}}{1+\kappa_{21}x_{7}}$ in ${\dot{x}}_{4}$ and $\frac{1+\rho_{12}x_{6}}{1+\kappa_{12}x_{6}}$ in ${\dot{x}}_{5}$ (with $\rho_{\cdot \cdot },\kappa_{\cdot \cdot }\;\geq 0$).
- *Switching between believers* $\left(x_{6}⇆x_{7}\right)$: add linear transfers $-\sigma_{67}x_{6}+\sigma_{76}x_{7}$ in ${\dot{x}}_{6}$ and $-\sigma_{76}x_{7}+\sigma_{67}x_{6}$ in ${\dot{x}}_{7}$ (with $\sigma_{67},\sigma_{76}\;\geq 0$).

**Expected impact:** For *small* cross-coefficients, the primary delay-driven stability boundary in τ and the qualitative picture we report are essentially unchanged; *larger* cross-effects can split or merge oscillatory windows and reshape transients, consistent with multi-rumor interaction studies [7,12]. We therefore keep the minimal core in the main analysis and leave calibrated cross-terms to future, platform-specific work.

**Proposition 1.**  *If the initial conditions are non-negative, the solution remains non-negative on its interval of existence.*

**Proof.**  The first equation gives

$$x_{1}\left(t\right)=e^{-\mu t-\alpha \int_{0}^{t}x_{2}\left(s\right)ds}\left[x_{1}\left(0\right)+\Lambda \int_{0}^{t}e^{\mu s+\alpha \int_{0}^{s}x_{2}\left(\theta \right)d\theta }ds\right]$$

and this is obviously positive when $x_{1}\left(0\right)\geq 0$. Next, the second equation gives

$$x_{2}\left(t\right)=x_{2}\left(0\right)e^{-\mu t+\int_{0}^{t}\left(\alpha x_{1}\left(s\right)-\eta x_{3}\left(s-\tau \right)\right)ds}$$

and $x_{2}\left(0\right)\geq 0$ implies the positivity of $x_{2}$.

It follows from (3) that $x_{3}\left(t\right)>0\;\forall \;t\in \left[0,t_{1}\right),x_{3}\left(t_{1}\right)=0\Rightarrow x^{\prime }\left(t_{1}\right)>0$ and this yields a contradiction.

Equation (4) gives

$$x_{4}\left(t\right)=e^{-\gamma_{1}t}\left[x_{4}\left(0\right)+\int_{0}^{t}\frac{\beta_{1}x_{2}\left(s\right)x_{6}\left(s\right)}{1+\alpha_{1}x_{2}\left(s\right)+\epsilon_{1}x_{6}\left(s\right)}e^{\gamma_{1}s}\;ds\right],$$

and from (6),

$$x_{6}\left(t\right)=e^{-\mu_{1}t}\left[x_{6}\left(0\right)+\int_{0}^{t}\gamma_{1}x_{4}\left(s\right)e^{\mu_{1}s}\;ds\right].$$

We see that $x_{4}\left(t\right)>0,\forall \;t\in \left[0,t_{1}\right)\Rightarrow x_{6}\left(t\right)>0\;\forall \;t\in \left[0,t_{1}\right)$ and since $x_{2}\left(t\right)>0\;\forall \;t,$ it cannot happen that $x_{4}\left(t_{1}\right)=0$. It follows that $x_{4}\left(0\right)>0\Rightarrow x_{4}\left(t\right)>0\;\forall \;t,x_{6}\left(0\right)>0\Rightarrow x_{6}\left(t\right)>0\;\forall \;t.$ The same reasoning applies to $x_{5},x_{7}$, so they are also positive on the whole domain of existence, and the positivity of $x_{8}$ follows. □

### 2.3. Equilibria

An equilibrium $E=(x_{1}^{*},x_{2}^{*},x_{3}^{*},x_{4}^{*},x_{5}^{*},x_{6}^{*},x_{7}^{*},x_{8}^{*})$ satisfies ${\dot{x}}_{i}=0$ for all *i*. We note the following types:

**-$E_{1}$ (rumor-free equilibrium):** This equilibrium represents a scenario where no active rumors spreaders exist; that is, all variables related to the rumor process ($x_{2},x_{3},x_{4},\ldots ,x_{8}$) are zero. If $E_{1}$ is stable, it implies that the rumors naturally disappear from the system.

**-$E_{2}$ (endemic equilibrium):** This equilibrium describes a situation where rumors persist in the system. In this case, the variables $x_{1},x_{2},x_{3},x_{4},\ldots ,x_{8}$ are nonzero, meaning that the spread of rumors continues, possibly in a stable way. If $E_{2}$ is stable, then rumors remain active indefinitely.

**-**$E_{3}.$ This equilibrium reflects a situation when the first rumor dies out, but the second persists, so the variables $x_{1},x_{2},x_{3},x_{5},x_{7},x_{8}$ are nonzero, while $x_{4}=x_{6}=0$

**-**$E_{4}.$ This equilibrium reflects a situation when the second rumor dies out but the first one persists, so the variables $x_{1},x_{2},x_{3},x_{4},x_{6},x_{8}$ are nonzero while $x_{5}=x_{7}=0$

**-**$E_{5}.$ This is an equilibrium that describes the situation when both rumors die out but people continue to be active on OSM. Then the variables $x_{1},x_{2},x_{3}$ are nonzero and $x_{4}=x_{5}=x_{6}=x_{7}=x_{8}=0$.

**Rumor-free equilibrium $E_{1}$**

Setting $x_{2}^{*}=x_{3}^{*}=x_{4}^{*}=\cdots =x_{8}^{*}=0$ in the system, we obtain

$$0\;=\;\Lambda -\alpha \;x_{1}^{*}\cdot 0\;-\;\mu \;x_{1}^{*}\;⟹\;\Lambda =\mu \;x_{1}^{*}\;⟹\;x_{1}^{*}=\frac{\Lambda }{\mu }.$$

Hence,

$$E_{1}=\left(\frac{\Lambda }{\mu },\;0,\;0,\;0,\;0,\;0,\;0,\;0\right).$$

**Endemic (rumor-present) equilibrium $E_{2}$**

If $x_{2}^{*}>0$ and the other rumor-related compartments are also potentially positive, the equilibrium conditions are as follows:

$$\left\{\begin{array}{c} 0=\Lambda -\alpha \;x_{1}^{*}\;x_{2}^{*}-\mu \;x_{1}^{*},\\ 0=\alpha \;x_{1}^{*}-\eta \;x_{3}^{*}-\mu ,\\ 0=\eta \;x_{2}^{*}\;x_{3}^{*}-\mu \;x_{3}^{*}+\omega \;x_{8}^{*},\\ 0=\frac{\beta_{1}\;x_{2}^{*}\;x_{6}^{*}}{\;1+\alpha_{1}\;x_{2}^{*}+\epsilon_{1}\;x_{6}^{*}\;}-\gamma_{1}\;x_{4}^{*},\\ 0=\frac{\beta_{2}\;x_{2}^{*}\;x_{7}^{*}}{\;1+\alpha_{2}\;x_{2}^{*}+\epsilon_{2}\;x_{7}^{*}\;}-\gamma_{2}\;x_{5}^{*},\\ 0=\gamma_{1}\;x_{4}^{*}-\mu_{1}\;x_{6}^{*},\\ 0=\gamma_{2}\;x_{5}^{*}-\mu_{2}\;x_{7}^{*},\\ 0=\mu_{1}\;x_{6}^{*}+\mu_{2}\;x_{7}^{*}-\omega \;x_{8}^{*}. \end{array}\right.$$

From these equations, we obtain the following:

$$x_{1}^{*}=\frac{\Lambda }{\alpha x_{2}^{*}+\mu },\;x_{3}^{*}=\frac{\alpha x_{1}^{*}-\mu }{\eta },\;x_{4}^{*}=\frac{\mu_{1}}{\gamma_{1}}x_{6}^{*},\;x_{5}^{*}=\frac{\mu_{2}}{\gamma_{2}}x_{7}^{*},$$

$$x_{6}^{*}=\frac{\left(\beta_{1}-\alpha_{1}\mu_{1}\right)x_{2}^{*}-\mu_{1}}{\epsilon_{1}\mu_{1}},\;x_{7}^{*}=\frac{\left(\beta_{2}-\alpha_{2}\mu_{2}\right)x_{2}^{*}-\mu_{2}}{\epsilon_{2}\mu_{2}}.$$

The last equilibrium equation directly gives

$$\omega x_{8}^{*}=\mu_{1}x_{6}^{*}+\mu_{2}x_{7}^{*}.$$

Substituting this relation into the third equilibrium equation removes ω entirely.

$$S_{\beta }=\frac{\beta_{1}-\alpha_{1}\mu_{1}}{\epsilon_{1}}+\frac{\beta_{2}-\alpha_{2}\mu_{2}}{\epsilon_{2}},\;S_{\mu }=\frac{\mu_{1}}{\epsilon_{1}}+\frac{\mu_{2}}{\epsilon_{2}}.$$

Let $x:=x_{2}^{*}$. The endemicity condition reduces to

(9) $$Ax^{2}+Bx+C=0,$$

with

$$A=\alpha \;\left(S_{\beta }-\mu \right),\;B=\alpha \Lambda +\left(\alpha -1\right)\mu^{2}+\mu \;\left(\frac{\beta_{1}-\alpha_{1}\mu_{1}}{\epsilon_{1}}+\frac{\beta_{2}-\alpha_{2}\mu_{2}}{\epsilon_{2}}\right),\;C=-\alpha \Lambda \;\mu +\mu^{3}-\mu \eta \;S_{\mu }.$$

A positive endemic solution exists if

$$\Delta :=B^{2}-4AC\geq 0,$$

and the chosen root satisfies

$$x_{2}^{*}=\frac{-B+\sqrt{\Delta }}{2A}>0.$$

$$x_{6}^{*}=\frac{\left(\beta_{1}-\alpha_{1}\mu_{1}\right)\;x_{2}^{*}-\mu_{1}}{\epsilon_{1}\mu_{1}}\geq 0\;⟹\;\left(\beta_{1}-\alpha_{1}\mu_{1}\right)\;x_{2}^{*}\geq \mu_{1},$$

$$x_{7}^{*}=\frac{\left(\beta_{2}-\alpha_{2}\mu_{2}\right)\;x_{2}^{*}-\mu_{2}}{\epsilon_{2}\mu_{2}}\geq 0\;⟹\;\left(\beta_{2}-\alpha_{2}\mu_{2}\right)\;x_{2}^{*}\geq \mu_{2},$$

with $\epsilon_{1},\epsilon_{2}>0$.

**Positivity constraints for believer classes:** From

$$x_{6}^{*}=\frac{\left(\beta_{1}-\alpha_{1}\mu_{1}\right)\;x_{2}^{*}-\mu_{1}}{\epsilon_{1}\mu_{1}},\;x_{7}^{*}=\frac{\left(\beta_{2}-\alpha_{2}\mu_{2}\right)\;x_{2}^{*}-\mu_{2}}{\epsilon_{2}\mu_{2}},$$

the necessary and sufficient conditions for $x_{6}^{*},x_{7}^{*}\geq 0$ are

$$\begin{array}{c} \;\beta_{1}x_{2}^{*}\geq \mu_{1}\left(1+\alpha_{1}x_{2}^{*}\right)\; \end{array},\;\begin{array}{c} \;\beta_{2}x_{2}^{*}\geq \mu_{2}\left(1+\alpha_{2}x_{2}^{*}\right)\; \end{array}.$$

### 2.4. Analysis of Positivity Condition for $x_{3}^{*}$ in the Endemic Equilibrium $E_{2}$

In the endemic equilibrium $E_{2}$, we derive the expression for $x_{3}^{*}$ as follows:

$$x_{3}^{*}=\frac{\alpha x_{1}^{*}-\mu }{\eta }$$

For this equilibrium to be sociologically meaningful, we need $x_{3}^{*}>0$, which implies

$$\alpha x_{1}^{*}-\mu >0\;\Rightarrow \;x_{1}^{*}>\frac{\mu }{\alpha }$$

For $x_{3}^{*}$ to be positive, we need the following:

(10) $$x_{1}^{*}>\frac{\mu }{\alpha }.$$

The inequality (10) represents the condition for the positivity of $x_{3}^{*}$ in terms of the system parameters. The analysis shows that the positivity of $x_{3}^{*}$ depends on a delicate balance between the birth rate Λ, the interaction rates, and the death/removal rates in the system.

**Equilibrium $E_{3}$: (Rumor 1 dies; Rumor 2 persists).**

For equilibrium $E_{3}$, we have $x_{4}^{*}=x_{6}^{*}=0$ while $x_{5}^{*},x_{7}^{*}>0$. Therefore,

$$x_{1}^{*}=\frac{\Lambda }{\alpha \;x_{2}^{*}+\mu },\;x_{3}^{*}=\frac{\alpha \;x_{1}^{*}-\mu }{\eta },\;x_{5}^{*}=\frac{\mu_{2}}{\gamma_{2}}\;x_{7}^{*},\;x_{7}^{*}=\frac{\beta_{2}\;x_{2}^{*}-\alpha_{2}\;\mu_{2}\;x_{2}^{*}-\mu_{2}}{\epsilon_{2}\;\mu_{2}},\;x_{8}^{*}=\frac{\mu_{2}\;x_{7}^{*}}{\omega }$$

**Equilibrium $E_{4}$: (Rumor 2 dies; Rumor 1 persists).** We have $x_{5}^{*}=x_{7}^{*}=0$ while $x_{4}^{*},x_{6}^{*}>0$; therefore,

$$x_{1}^{*}=\frac{\Lambda }{\alpha \;x_{2}^{*}+\mu },\;x_{3}^{*}=\frac{\alpha \;x_{1}^{*}-\mu }{\eta },\;x_{4}^{*}=\frac{\mu_{1}}{\gamma_{1}}\;x_{6}^{*},\;x_{6}^{*}=\frac{\beta_{1}\;x_{2}^{*}-\alpha_{1}\;\mu_{1}\;x_{2}^{*}-\mu_{1}}{\epsilon_{1}\;\mu_{1}},\;x_{8}^{*}=\frac{\mu_{1}\;x_{6}^{*}}{\omega }$$

**Equilibrium $E_{5}$: Both rumors die out, but individuals remain active.** Setting $x_{4}^{*}=x_{5}^{*}=x_{6}^{*}=x_{7}^{*}=x_{8}^{*}=0$ in the system of equations, we can derive the expressions for $x_{1}^{*},x_{2}^{*},x_{3}^{*}$.

Thus, the equilibrium point $E_{5}$ is as follows:

$$E_{5}=\left(\frac{\Lambda \eta }{\mu (\alpha +\eta )},\frac{\mu }{\eta },\frac{\alpha \left(\frac{\Lambda \eta }{\mu (\alpha +\eta )}\right)-\mu }{\eta },0,0,0,0,0\right)$$

### 2.5. Analysis of Positivity Condition for $x_{3}^{*}$ in the Endemic Equilibrium $E_{5}$

For $E_{5}$ to be sociologically meaningful, all its components must be positive. We already have $x_{1}^{*}>0$ and $x_{2}^{*}>0$, given the positivity of the parameters. For $x_{3}^{*}>0$, we need

$$\frac{\alpha x_{1}^{*}-\mu }{\eta }>0\;\Rightarrow \;\alpha x_{1}^{*}-\mu >0\;\Rightarrow \;\alpha x_{1}^{*}>\mu$$

Substituting the expression for $x_{1}^{*}$,

$$\alpha \frac{\Lambda \eta }{\mu (\alpha +\eta )}>\mu \;\Rightarrow \;\alpha \Lambda \eta >\mu^{2}\left(\alpha +\eta \right)$$

This condition determines the existence of a positive $x_{3}^{*}$ for $E_{5}$.

## 3. Stability Conditions for the Equilibrium Points

In all the equilibrium points $E_{2},\ldots ,E_{5}$. $x_{2}^{*}\neq 0$ and $x_{2}^{*}$ is calculated as a root of a second-degree equation that can have zero, one, or two solutions.

- For our system, we study the stability of the equilibrium points $E_{1},E_{2},E_{3},E_{4},E_{5}$,when they exist. We denote the following:
- $\frac{\partial f}{\partial x}$ The matrix of partial derivatives with respect to the non-delayed variables, evaluated at the equilibrium;
- $\frac{\partial f}{\partial x_{\tau }}$ The matrix of partial derivatives with respect to the delayed variable (here, $x_{3}\left(t-\tau \right)$).

### 3.1. Characteristic Equations for All Computed Equilibrium Points

To analyze stability at the first equilibrium point $E_{1}$, we consider the characteristic equation:

$$\det\left(\lambda I-\frac{\partial f}{\partial x}-\frac{\partial f}{\partial x_{\tau }}e^{-\lambda \tau }\right)=0.$$

Recall $E_{1}=\left(\frac{\Lambda }{\mu },\;0,\;0,\;0,\;0,\;0,\;0,\;0\right).$ Substituting $x_{1}^{*}=\Lambda /\mu$ and $x_{2}^{*}=\cdots =x_{8}^{*}=0$, we obtain

$$\frac{\partial f}{\partial x}{|}_{E_{1}}=\left(\begin{array}{cccccccc} -\mu & -\alpha \;\frac{\Lambda }{\mu } & 0 & 0 & 0 & 0 & 0 & 0\\ 0 & \alpha \;\frac{\Lambda }{\mu }-\mu & 0 & 0 & 0 & 0 & 0 & 0\\ 0 & 0 & -\mu & 0 & 0 & 0 & 0 & \omega \\ 0 & 0 & 0 & -\gamma_{1} & 0 & 0 & 0 & 0\\ 0 & 0 & 0 & 0 & -\gamma_{2} & 0 & 0 & 0\\ 0 & 0 & 0 & \gamma_{1} & 0 & -\mu_{1} & 0 & 0\\ 0 & 0 & 0 & 0 & \gamma_{2} & 0 & -\mu_{2} & 0\\ 0 & 0 & 0 & 0 & 0 & \mu_{1} & \mu_{2} & -\omega \end{array}\right)$$

Since only (2) and (3) depend on the delayed state, the delayed Jacobian $\frac{\partial f}{\partial x_{\tau }}$ is zero at $E_{1}$:

$$\left|\begin{array}{cccccccc} \lambda +\mu & \alpha \frac{\Lambda }{\mu } & 0 & 0 & 0 & 0 & 0 & 0\\ 0 & \lambda -\left(\alpha \frac{\Lambda }{\mu }-\mu \right) & 0 & 0 & 0 & 0 & 0 & 0\\ 0 & 0 & \lambda +\mu & 0 & 0 & 0 & 0 & -\omega \\ 0 & 0 & 0 & \lambda +\gamma_{1} & 0 & 0 & 0 & 0\\ 0 & 0 & 0 & 0 & \lambda +\gamma_{2} & 0 & 0 & 0\\ 0 & 0 & 0 & -\gamma_{1} & 0 & \lambda +\mu_{1} & 0 & 0\\ 0 & 0 & 0 & 0 & -\gamma_{2} & 0 & \lambda +\mu_{2} & 0\\ 0 & 0 & 0 & 0 & 0 & -\mu_{1} & -\mu_{2} & \lambda +\omega \end{array}\right|=0$$

The equation becomes

(11) $${\left(\lambda +\mu \right)}^{2}\left(\lambda -a_{22}\right)\left(\lambda +\gamma_{1}\right)\left(\lambda +\gamma_{2}\right)\left(\lambda +\mu_{1}\right)\left(\lambda +\mu_{2}\right)\left(\lambda +\omega \right)=0$$

### 3.2. Stability Condition for $E_{1}$

**Theorem 1**  (Stability of $E_{1}$). *The rumor-free equilibrium $E_{1}=\left(\frac{\Lambda }{\mu },0,0,0,0,0,0,0\right)$ is locally asymptotically stable if and only if $\mu^{2}>\alpha \Lambda$.*

**Proof.**  To establish the stability of $E_{1}$, we must analyze the complete linearized system around this equilibrium point. The stability is determined by the eigenvalues of the Jacobian matrix evaluated at $E_{1}$.

These eigenvalues are as follows:

$$\lambda_{1}=\lambda_{2}=-\mu <0,\;\lambda_{3}=\alpha \frac{\Lambda }{\mu }-\mu ,\;\lambda_{4}=-\omega <0,$$

$$\lambda_{5}=-\gamma_{1}<0,\;\lambda_{6}=-\mu_{1}<0,\;\lambda_{7}=-\gamma_{2}<0,\;\lambda_{8}=-\mu_{2}<0$$

For local asymptotic stability, all eigenvalues must have negative real parts. All eigenvalues are negative except possibly λ=αΛ/μ−μ, so the stability condition reduces to

$$\lambda_{2}=\alpha \frac{\Lambda }{\mu }-\mu <0$$

This is equivalent to $\mu^{2}>\alpha \Lambda ;$ therefore, $E_{1}$ is locally asymptotically stable if and only if $\mu^{2}>\alpha \Lambda$. □

#### Sociological Interpretation

The condition $\mu^{2}>\alpha \Lambda$ means that the rate at which individuals leave the system, squared, must exceed the rate at which potential users become active and are exposed to new information. When this condition fails ($\mu^{2}\leq \alpha \Lambda$), the rumor-free equilibrium becomes unstable, and rumors will inevitably spread in the population.

## 4. Stability Analysis of Endemic Equilibrium Point $E_{2}$

To analyze the stability of this equilibrium point, we compute the Jacobian matrix of the system. The partial derivatives are as follows:

$$\begin{array}{cc} & a_{11}=\frac{\partial f_{1}}{\partial x_{1}}=-\alpha x_{2}^{*}-\mu ,\;a_{12}=\frac{\partial f_{1}}{\partial x_{2}}=-\alpha x_{1}^{*},\\ & a_{21}=\frac{\partial f_{2}}{\partial x_{1}}=\alpha x_{2}^{*},\;a_{22}=\frac{\partial f_{2}}{\partial x_{2}}=\alpha x_{1}^{*}-\eta x_{3}^{*}-\mu ,\;a_{23}=\frac{\partial f_{2}}{\partial x_{3}}=0,\\ & a_{32}=\frac{\partial f_{3}}{\partial x_{2}}=\eta x_{3}^{*},\;a_{33}=\frac{\partial f_{3}}{\partial x_{3}}=-\mu ,\;a_{38}=\frac{\partial f_{3}}{\partial x_{8}}=\omega ,\\ & a_{42}=\frac{\partial f_{4}}{\partial x_{2}}=\frac{\beta_{1}x_{6}^{*}}{{\left(1+\alpha_{1}x_{2}^{*}+\epsilon_{1}x_{6}^{*}\right)}^{2}},\;a_{44}=\frac{\partial f_{4}}{\partial x_{4}}=-\gamma_{1},\;a_{46}=\frac{\partial f_{4}}{\partial x_{6}}=\frac{\beta_{1}x_{2}^{*}}{{\left(1+\alpha_{1}x_{2}^{*}+\epsilon_{1}x_{6}^{*}\right)}^{2}},\\ & a_{52}=\frac{\partial f_{5}}{\partial x_{2}}=\frac{\beta_{2}x_{7}^{*}}{{\left(1+\alpha_{2}x_{2}^{*}+\epsilon_{2}x_{7}^{*}\right)}^{2}},\;a_{55}=\frac{\partial f_{5}}{\partial x_{5}}=-\gamma_{2},\;a_{57}=\frac{\partial f_{5}}{\partial x_{7}}=\frac{\beta_{2}x_{2}^{*}}{{\left(1+\alpha_{2}x_{2}^{*}+\epsilon_{2}x_{7}^{*}\right)}^{2}},\\ & a_{64}=\frac{\partial f_{6}}{\partial x_{4}}=\gamma_{1},\;a_{66}=\frac{\partial f_{6}}{\partial x_{6}}=-\mu_{1},\;a_{75}=\frac{\partial f_{7}}{\partial x_{5}}=\gamma_{2},\;a_{77}=\frac{\partial f_{7}}{\partial x_{7}}=-\mu_{2},\\ & a_{86}=\frac{\partial f_{8}}{\partial x_{6}}=\mu_{1},\;a_{87}=\frac{\partial f_{8}}{\partial x_{7}}=\mu_{2},\;a_{88}=\frac{\partial f_{8}}{\partial x_{8}}=-\omega . \end{array}$$

The Jacobian matrix $\frac{\partial f}{\partial x},$ evaluated at $E_{2}$ (with state order $x_{1},x_{2},\ldots ,x_{8}$), is as follows:

$$\frac{\partial f}{\partial x}{|}_{E_{2}}=\left(\begin{array}{cccccccc} -\alpha \;x_{2}-\mu & -\alpha \;x_{1} & 0 & 0 & 0 & 0 & 0 & 0\\ \alpha \;x_{2} & \alpha \;x_{1}-\eta \;x_{3}-\mu & 0 & 0 & 0 & 0 & 0 & 0\\ 0 & \eta \;x_{3} & -\mu & 0 & 0 & 0 & 0 & \omega \\ 0 & \frac{\beta_{1}\;x_{6}\left(1+\epsilon_{1}\;x_{6}\right)}{D_{1}^{2}} & 0 & -\gamma_{1} & 0 & \frac{\beta_{1}\;x_{2}\left(1+\alpha_{1}\;x_{2}\right)}{D_{1}^{2}} & 0 & 0\\ 0 & \frac{\beta_{2}\;x_{7}\left(1+\epsilon_{2}\;x_{7}\right)}{D_{2}^{2}} & 0 & 0 & -\gamma_{2} & 0 & \frac{\beta_{2}\;x_{2}\left(1+\alpha_{2}\;x_{2}\right)}{D_{2}^{2}} & 0\\ 0 & 0 & 0 & \gamma_{1} & 0 & -\mu_{1} & 0 & 0\\ 0 & 0 & 0 & 0 & \gamma_{2} & 0 & -\mu_{2} & 0\\ 0 & 0 & 0 & 0 & 0 & \mu_{1} & \mu_{2} & -\omega \end{array}\right)$$

where

$$D_{1}=1+\alpha_{1}\;x_{2}+\epsilon_{1}\;x_{6},\;D_{2}=1+\alpha_{2}\;x_{2}+\epsilon_{2}\;x_{7}.$$

Only Equations (2) and (3) depend on the delayed variable $x_{3}\left(t-\tau \right)$. The derivatives are

$$b_{23}=\frac{\partial f_{2}}{\partial x_{3\tau }}=-\eta \;x_{2},\;b_{33}=\frac{\partial f_{3}}{\partial x_{3\tau }}=\eta \;x_{2}.$$

Thus, the Jacobian matrix with respect to delayed terms, evaluated at $E_{2}$, is as follows:

$$\frac{\partial f}{\partial x_{\tau }}{|}_{E_{2}}=\left(\begin{array}{cccccccc} 0 & 0 & 0 & 0 & 0 & 0 & 0 & 0\\ 0 & 0 & -\eta \;x_{2} & 0 & 0 & 0 & 0 & 0\\ 0 & 0 & \eta \;x_{2} & 0 & 0 & 0 & 0 & 0\\ 0 & 0 & 0 & 0 & 0 & 0 & 0 & 0\\ 0 & 0 & 0 & 0 & 0 & 0 & 0 & 0\\ 0 & 0 & 0 & 0 & 0 & 0 & 0 & 0\\ 0 & 0 & 0 & 0 & 0 & 0 & 0 & 0\\ 0 & 0 & 0 & 0 & 0 & 0 & 0 & 0 \end{array}\right).$$

The characteristic equation for the linearized system around $E_{2}$ with delay is

(12) $$\det(\lambda I-A-Be^{-\lambda \tau })=0$$

where *A* is the 8×8 Jacobian matrix, and *B* is the 8×8 delay matrix. Explicitly, this determinant is as follows:

(13) $$\left|\begin{array}{cccccccc} \lambda -a_{11} & -a_{12} & 0 & 0 & 0 & 0 & 0 & 0\\ -a_{21} & \lambda -a_{22} & -b_{23}e^{-\lambda \tau } & 0 & 0 & 0 & 0 & 0\\ 0 & -a_{32} & \lambda -a_{33}-b_{33}e^{-\lambda \tau } & 0 & 0 & 0 & 0 & -a_{38}\\ 0 & -a_{42} & 0 & \lambda -a_{44} & 0 & -a_{46} & 0 & 0\\ 0 & -a_{52} & 0 & 0 & \lambda -a_{55} & 0 & -a_{57} & 0\\ 0 & 0 & 0 & -a_{64} & 0 & \lambda -a_{66} & 0 & 0\\ 0 & 0 & 0 & 0 & -a_{75} & 0 & \lambda -a_{77} & 0\\ 0 & 0 & 0 & 0 & 0 & -a_{86} & -a_{87} & \lambda -a_{88} \end{array}\right|=0$$

The presence of $a_{42},a_{52}$ on column 2 of the determinant makes this equation difficult to handle. We apply the rank-one perturbation argument from [16]: if the sum $|a_{42}\left|+\right|a_{52}|$ is small, in a sense made precise in [16], then, if the equation

(14) $$\left|\begin{array}{cccccccc} \lambda -a_{11} & -a_{12} & 0 & 0 & 0 & 0 & 0 & 0\\ -a_{21} & \lambda -a_{22} & -b_{23}e^{-\lambda \tau } & 0 & 0 & 0 & 0 & 0\\ 0 & -a_{32} & \lambda -a_{33}-b_{33}e^{-\lambda \tau } & 0 & 0 & 0 & 0 & -a_{38}\\ 0 & 0 & 0 & \lambda -a_{44} & 0 & -a_{46} & 0 & 0\\ 0 & 0 & 0 & 0 & \lambda -a_{55} & 0 & -a_{57} & 0\\ 0 & 0 & 0 & -a_{64} & 0 & \lambda -a_{66} & 0 & 0\\ 0 & 0 & 0 & 0 & -a_{75} & 0 & \lambda -a_{77} & 0\\ 0 & 0 & 0 & 0 & 0 & -a_{86} & -a_{87} & \lambda -a_{88} \end{array}\right|=0$$

has only roots with negative real parts, the same will be true for the perturbed Equation (13). Equation (14) factors into the following:

(15) $$d_{1}=\left|\begin{array}{ccc} \lambda -a_{11} & -a_{12} & 0\\ -a_{21} & \lambda -a_{22} & -b_{23}e^{-\lambda \tau }\\ 0 & -a_{32} & \lambda -a_{33}-b_{33}e^{-\lambda \tau } \end{array}\right|=0$$

and

(16) $$d_{2}=\left|\begin{array}{ccccc} \lambda -a_{44} & 0 & -a_{46} & 0 & 0\\ 0 & \lambda -a_{55} & 0 & -a_{57} & 0\\ -a_{64} & 0 & \lambda -a_{66} & 0 & 0\\ 0 & -a_{75} & 0 & \lambda -a_{77} & 0\\ 0 & 0 & -a_{86} & -a_{87} & \lambda -a_{88} \end{array}\right|=0$$

As is well known (see, for example, [17]), if the equilibrium is stable for τ=0, stability can be lost if the imaginary axis is crossed, i.e., when τ varies, from left to right. So, we study if λ=ir (see [17]) can be a root of (15) and, when this is the case, we calculate the derivative of λ with respect to τ. The equation $d_{2}=0$ is equivalent to $\left(\lambda +\omega \right)d_{3}=0$ where

(17) $$d_{3}=\left|\begin{array}{cccc} \lambda +\gamma_{1} & 0 & -a_{46} & 0\\ 0 & \lambda +\gamma_{2} & 0 & -a_{57}\\ -\gamma_{1} & 0 & \lambda +\mu_{1} & 0\\ 0 & -\gamma_{2} & 0 & \lambda +\mu_{2} \end{array}\right|=0$$

Using the Schur complement argument, one obtains

$$d_{3}=\left[\lambda^{2}+\lambda \left(\gamma_{1}+\mu_{1}\right)+\gamma_{1}\left(\mu_{1}-a_{46}\right)\right]\left[\lambda^{2}+\lambda \left(\gamma_{2}+\mu_{2}\right)+\gamma_{2}\left(\mu_{2}-a_{57}\right)\right]=0$$

and in order to have stability, it is sufficient that

(18) $$\mu_{1}>a_{46},\mu_{2}>a_{57}$$

After some calculations, Equation (15) becomes

(19) $$\begin{array}{cc} d_{1} & =\left(\lambda -a_{11}\right)\left[\left(\lambda -a_{22}\right)\left(\lambda -a_{33}-b_{33}e^{-\lambda \tau }\right)-\left(-a_{23}-b_{23}e^{-\lambda \tau }\right)\left(-a_{32}\right)\right]-\left(-a_{12}\right)\left[\left(-a_{21}\right)\left(\lambda -a_{33}-b_{33}e^{-\lambda \tau }\right)\right]\\ \; & =\left(\lambda -a_{11}\right)\left[\left(\lambda -a_{22}\right)\left(\lambda -a_{33}\right)-\left(\lambda -a_{22}\right)b_{33}e^{-\lambda \tau }-a_{32}\left(a_{23}+b_{23}e^{-\lambda \tau }\right)\right]+a_{12}\left[-a_{21}\left(\lambda -a_{33}\right)-a_{21}\left(-b_{33}e^{-\lambda \tau }\right)\right]\\ \; & =\left(\lambda -a_{11}\right)\left[\left(\lambda -a_{22}\right)\left(\lambda -a_{33}\right)-a_{23}a_{32}-\left(\lambda -a_{22}\right)b_{33}e^{-\lambda \tau }-a_{32}b_{23}e^{-\lambda \tau }\right]-a_{12}a_{21}\left(\lambda -a_{33}\right)+a_{12}a_{21}b_{33}e^{-\lambda \tau } \end{array}$$

We separate the terms into P(λ) (terms without $e^{-\lambda \tau }$) and Q(λ) (coefficients of $e^{-\lambda \tau }$):

$$\begin{array}{cc} P(\lambda ) & =\left(\lambda -a_{11}\right)\left[\left(\lambda -a_{22}\right)\left(\lambda -a_{33}\right)-a_{23}a_{32}\right]-a_{12}a_{21}\left(\lambda -a_{33}\right)\\ Q(\lambda ) & =-\left(\lambda -a_{11}\right)\left[\left(\lambda -a_{22}\right)b_{33}+a_{32}b_{23}\right]+a_{12}a_{21}b_{33} \end{array}$$

The characteristic equation is then $P\left(\lambda \right)+Q\left(\lambda \right)e^{-\lambda \tau }=0$.

### 4.1. Stability Analysis When τ=0

When τ=0, the characteristic equation simplifies to the following:

$$\begin{array}{cc} P(\lambda )+Q(\lambda ) & =\left(\lambda -a_{11}\right)\left[\left(\lambda -a_{22}\right)\left(\lambda -a_{33}\right)-a_{23}a_{32}\right]-a_{12}a_{21}\left(\lambda -a_{33}\right)-\left(\lambda -a_{11}\right)\left[\left(\lambda -a_{22}\right)b_{33}+a_{32}b_{23}\right]+a_{12}a_{21}b_{33}\\ \; & =\left(\lambda -a_{11}\right)\left[\left(\lambda -a_{22}\right)\left(\lambda -a_{33}\right)-a_{23}a_{32}-\left(\lambda -a_{22}\right)b_{33}-a_{32}b_{23}\right]-a_{12}a_{21}\left(\lambda -a_{33}-b_{33}\right)=0 \end{array}$$

Expanding and collecting the terms by powers of λ, we obtain the polynomial

$$\lambda^{3}+A_{1}\lambda^{2}+A_{2}\lambda +A_{3}=0,\;\mathrm{where}\;\mathrm{the}\;\mathrm{coefficients}\;\mathrm{are}\;\mathrm{as}\;\mathrm{follows}:$$

-
$A_{1}=-\left(a_{11}+a_{22}+a_{33}+b_{33}\right)$

-
$A_{2}=\left(a_{22}a_{33}-a_{23}a_{32}+a_{22}b_{33}-a_{32}b_{23}\right)+a_{11}\left(a_{22}+a_{33}+b_{33}\right)-a_{12}a_{21}$

-
$A_{3}=-\left[a_{11}\left(a_{22}a_{33}-a_{23}a_{32}+a_{22}b_{33}-a_{32}b_{23}\right)-a_{12}a_{21}\left(a_{33}+b_{33}\right)\right]$

For a cubic polynomial, the Routh–Hurwitz stability criteria state that all roots have negative real parts if and only if

1.
$A_{1}>0$

2.
$A_{3}>0$

3.
$A_{1}A_{2}-A_{3}>0$

If these conditions are satisfied, then the endemic equilibrium point $E_{2}$ is locally asymptotically stable when the delay τ=0.

### 4.2. Stability Analysis for Endemic Equilibrium Point $E_{2}$ When τ>0

When τ>0, we substitute λ=ir as in [18] into the characteristic Equation (19), where *r* is a real positive number.

First, let us express P(ir) and Q(ir) in terms of their real and imaginary parts.

For $P\left(\lambda \right)=\left(\lambda -a_{11}\right)\left[\left(\lambda -a_{22}\right)\left(\lambda -a_{33}\right)-a_{23}a_{32}\right]-a_{12}a_{21}\left(\lambda -a_{33}\right)$:

$$\begin{array}{cc} P(ir) & =\left(ir-a_{11}\right)\left[\left(ir-a_{22}\right)\left(ir-a_{33}\right)-a_{23}a_{32}\right]-a_{12}a_{21}\left(ir-a_{33}\right)\\ \; & =\left(ir-a_{11}\right)\left[-r^{2}-i\left(a_{22}+a_{33}\right)r+a_{22}a_{33}-a_{23}a_{32}\right]-a_{12}a_{21}ir+a_{12}a_{21}a_{33}\\ \; & =ir\left(-r^{2}+a_{22}a_{33}-a_{23}a_{32}\right)+r\left(a_{22}+a_{33}\right)r-a_{11}\left(-r^{2}+a_{22}a_{33}-a_{23}a_{32}\right)+\\ & +ia_{11}\left(a_{22}+a_{33}\right)r-a_{12}a_{21}ir+a_{12}a_{21}a_{33}\\ \; & =[r^{2}\left(a_{22}+a_{33}\right)+a_{11}r^{2}-a_{11}\left(a_{22}a_{33}-a_{23}a_{32}\right)+a_{12}a_{21}a_{33}]\\ \; & \;+i[-r^{3}+r\left(a_{22}a_{33}-a_{23}a_{32}\right)+a_{11}\left(a_{22}+a_{33}\right)r-a_{12}a_{21}r] \end{array}$$

So, the real and imaginary parts of P(ir) are as follows:

$$\begin{array}{cc} Re(P(ir)) & =r^{2}\left(a_{11}+a_{22}+a_{33}\right)-a_{11}\left(a_{22}a_{33}-a_{23}a_{32}\right)+a_{12}a_{21}a_{33}\\ Im(P(ir)) & =-r^{3}+r\left(a_{22}a_{33}-a_{23}a_{32}+a_{11}a_{22}+a_{11}a_{33}-a_{12}a_{21}\right) \end{array}$$

Now, for $Q\left(\lambda \right)=-\left(\lambda -a_{11}\right)\left[\left(\lambda -a_{22}\right)b_{33}+a_{32}b_{23}\right]+a_{12}a_{21}b_{33}$,

$$\begin{array}{cc} Q(ir) & =-\left(ir-a_{11}\right)\left[\left(ir-a_{22}\right)b_{33}+a_{32}b_{23}\right]+a_{12}a_{21}b_{33}\\ \; & =-\left(ir-a_{11}\right)\left[irb_{33}-a_{22}b_{33}+a_{32}b_{23}\right]+a_{12}a_{21}b_{33}\\ \; & =-\left[ir\left(irb_{33}-a_{22}b_{33}+a_{32}b_{23}\right)-a_{11}\left(irb_{33}-a_{22}b_{33}+a_{32}b_{23}\right)\right]+a_{12}a_{21}b_{33}\\ \; & =-\left[-r^{2}b_{33}-ir\left(a_{22}b_{33}-a_{32}b_{23}\right)-ia_{11}rb_{33}+a_{11}a_{22}b_{33}-a_{11}a_{32}b_{23}\right]+a_{12}a_{21}b_{33}\\ \; & =\left[r^{2}b_{33}-a_{11}a_{22}b_{33}+a_{11}a_{32}b_{23}\right]+i\left[r\left(a_{22}b_{33}-a_{32}b_{23}+a_{11}b_{33}\right)\right]+a_{12}a_{21}b_{33} \end{array}$$

So, the real and imaginary parts of Q(ir) are

$$\begin{array}{cc} Re(Q(ir)) & =r^{2}b_{33}-a_{11}a_{22}b_{33}+a_{11}a_{32}b_{23}+a_{12}a_{21}b_{33}\\ Im(Q(ir)) & =r(a_{22}b_{33}-a_{32}b_{23}+a_{11}b_{33}) \end{array}$$

To simplify the notations, Re(P(ir)) will be denoted as Re(P) and the same for the other three. The characteristic equation $P\left(\lambda \right)+Q\left(\lambda \right)e^{-\lambda \tau }=0$ becomes

(Re(P)+iIm(P))+(Re(Q)+iIm(Q))(cos(rτ)−isin(rτ))=0

Expanding this, we obtain

$$Re\left(P\right)+iIm\left(P\right)+Re\left(Q\right)\cos\left(r\tau \right)-iRe\left(Q\right)\sin\left(r\tau \right)+iIm\left(Q\right)\cos\left(r\tau \right)-i^{2}Im\left(Q\right)\sin\left(r\tau \right)=0$$

Re(P)+iIm(P)+Re(Q)cos(rτ)−iRe(Q)sin(rτ)+iIm(Q)cos(rτ)+Im(Q)sin(rτ)=0

Separating the real and imaginary parts, we obtain a system of two equations:

(20) $$\begin{array}{cc} Re(P)+Re(Q)\cos(r\tau )+Im(Q)\sin(r\tau ) & =0 \end{array}$$

(21) $$\begin{array}{cc} Im(P)-Re(Q)\sin(r\tau )+Im(Q)\cos(r\tau ) & =0 \end{array}$$

**Solving the system for cos(rτ) and sin(rτ):**

From Equations (20) and (21), we can write

(22) $$\begin{array}{cc} Re(Q)\cos(r\tau )+Im(Q)\sin(r\tau ) & =-Re(P) \end{array}$$

(23) $$\begin{array}{cc} Im(Q)\cos(r\tau )-Re(Q)\sin(r\tau ) & =-Im(P) \end{array}$$

Solving this linear system for cos(rτ) and sin(rτ):

(24) $$\cos\left(r\tau \right)=\frac{-Re(P)Re(Q)-Im(P)Im(Q)}{Re{\left(Q\right)}^{2}+Im{\left(Q\right)}^{2}}$$

(25) $$\sin\left(r\tau \right)=\frac{-Re(P)Im(Q)+Im(P)Re(Q)}{Re{\left(Q\right)}^{2}+Im{\left(Q\right)}^{2}}$$

Using the identity $\cos^{2}\left(r\tau \right)+\sin^{2}\left(r\tau \right)=1$, we can derive the equation for $r_{c},$ where $\lambda =ir_{c}$ is the crossing eigenvalue:

$$\begin{array}{cc} {\left(\frac{-Re(P)Re(Q)-Im(P)Im(Q)}{Re{\left(Q\right)}^{2}+Im{\left(Q\right)}^{2}}\right)}^{2}+{\left(\frac{-Re(P)Im(Q)+Im(P)Re(Q)}{Re{\left(Q\right)}^{2}+Im{\left(Q\right)}^{2}}\right)}^{2} & =1\\ {\left[-Re\left(P\right)Re\left(Q\right)-Im\left(P\right)Im\left(Q\right)\right]}^{2}+{\left[-Re\left(P\right)Im\left(Q\right)+Im\left(P\right)Re\left(Q\right)\right]}^{2} & ={\left[Re{\left(Q\right)}^{2}+Im{\left(Q\right)}^{2}\right]}^{2}\\ {\left[Re\left(P\right)Re\left(Q\right)+Im\left(P\right)Im\left(Q\right)\right]}^{2}+{\left[Re\left(P\right)Im\left(Q\right)-Im\left(P\right)Re\left(Q\right)\right]}^{2} & ={\left[Re{\left(Q\right)}^{2}+Im{\left(Q\right)}^{2}\right]}^{2} \end{array}$$

Expanding the left side,

$$\begin{array}{c} Re{\left(P\right)}^{2}Re{\left(Q\right)}^{2}+2Re\left(P\right)Re\left(Q\right)Im\left(P\right)Im\left(Q\right)+Im{\left(P\right)}^{2}Im{\left(Q\right)}^{2}+Re{\left(P\right)}^{2}Im{\left(Q\right)}^{2}-\\ 2Re\left(P\right)Im\left(Q\right)Im\left(P\right)Re\left(Q\right)+Im{\left(P\right)}^{2}Re{\left(Q\right)}^{2}={\left[Re{\left(Q\right)}^{2}+Im{\left(Q\right)}^{2}\right]}^{2} \end{array}$$

Simplifying, the cross terms cancel out:

$$Re{\left(P\right)}^{2}Re{\left(Q\right)}^{2}+Im{\left(P\right)}^{2}Im{\left(Q\right)}^{2}+Re{\left(P\right)}^{2}Im{\left(Q\right)}^{2}+Im{\left(P\right)}^{2}Re{\left(Q\right)}^{2}={\left[Re{\left(Q\right)}^{2}+Im{\left(Q\right)}^{2}\right]}^{2}$$

Factor out common terms:

$$\begin{array}{c} Re{\left(P\right)}^{2}\left(Re{\left(Q\right)}^{2}+Im{\left(Q\right)}^{2}\right)+Im{\left(P\right)}^{2}\left(Im{\left(Q\right)}^{2}+Re{\left(Q\right)}^{2}\right)={\left[Re{\left(Q\right)}^{2}+Im{\left(Q\right)}^{2}\right]}^{2}\\ \left(Re{\left(P\right)}^{2}+Im{\left(P\right)}^{2}\right)\left(Re{\left(Q\right)}^{2}+Im{\left(Q\right)}^{2}\right)={\left[Re{\left(Q\right)}^{2}+Im{\left(Q\right)}^{2}\right]}^{2} \end{array}$$

Assuming $Re{\left(Q\right)}^{2}+Im{\left(Q\right)}^{2}\neq 0$, we can divide by $\left(Re{\left(Q\right)}^{2}+Im{\left(Q\right)}^{2}\right)$ to obtain the equation for $r_{c}$:

(26) $$Re{\left(P\right)}^{2}+Im{\left(P\right)}^{2}=Re{\left(Q\right)}^{2}+Im{\left(Q\right)}^{2}$$

Once $r_{c}$ is found, the critical delay $\tau_{critic}$ can be calculated following the approach outlined in [18]; specifically, see Remark 2.1 and Equation (2.41) in that reference, where the formulation of $\tau_{c}$ is given as follows:

$$\tau_{c}=\left\{\begin{array}{cc} \frac{1}{r}\arctan\left(\frac{\sin(r\tau )}{\cos(r\tau )}\right) & \mathrm{if}\;\sin(r\tau )>0,\cos(r\tau )>0\\ \frac{1}{r}\cdot \frac{\pi }{2} & \mathrm{if}\;\sin(r\tau )=1,\cos(r\tau )=0\\ \frac{1}{r}\left[\pi +\arctan\left(\frac{\sin(r\tau )}{\cos(r\tau )}\right)\right] & \mathrm{if}\;\cos(r\tau )<0\\ \frac{1}{r}\cdot \frac{3\pi }{2} & \mathrm{if}\;\sin(r\tau )=-1,\cos(r\tau )=0\\ \frac{1}{r}\left[2\pi +\arctan\left(\frac{\sin(r\tau )}{\cos(r\tau )}\right)\right] & \mathrm{if}\;\sin(r\tau )<0,\cos(r\tau )>0 \end{array}\right.$$

### 4.3. Derivative of λ

The Cook–van den Driessche method, as outlined in [19], offers a useful approach for analyzing the stability switches that occur as the delay parameter varies. An important step in this method is to calculate the derivative of the characteristic equation with respect to the complex variable λ, evaluated at purely imaginary roots.

The sign of $\frac{d\lambda }{d\tau }$ can be calculated by formula (2.12) from [19]. The positive value of the derivative $\frac{d\lambda }{d\tau }$ at the critical point indicates that as τ increases beyond $\tau_{c}$, the real part of the dominant eigenvalue increases, making the system more unstable through a Hopf bifurcation.

### 4.4. Characteristic Equation for $E_{3}$ and $E_{4}$ Using Rank-One Perturbation

For equilibria $E_{3}$ and $E_{4}$, the characteristic equation can be analyzed using the rank-one perturbation argument, similar to how it was introduced for $E_{2}$.

For $E_{3}$, we have $x_{4}^{*}=x_{6}^{*}=0$. The terms $a_{42},a_{46},a_{64},a_{86}$ will be affected. Since $x_{6}^{*}=0$, the terms $a_{42}$ and $a_{46}$ become zero. The characteristic equation will still involve $d_{1}$ and a modified $d_{2}$. The stability of the system is then determined by the roots of both $d_{1}=0$ and $d_{2}=0$.
  (27) $$d_{1}=\left|\begin{array}{ccc} \lambda -a_{11} & -a_{12} & 0\\ -a_{21} & \lambda -a_{22} & -b_{23}e^{-\lambda \tau }\\ 0 & -a_{32} & \lambda -a_{33}-b_{33}e^{-\lambda \tau } \end{array}\right|=0$$

This is the same cubic characteristic equation $P\left(\lambda \right)+Q\left(\lambda \right)e^{-\lambda \tau }=0$ as derived for $E_{2}$, with P(λ) and Q(λ) defined as follows:

$$\begin{array}{cc} P(\lambda ) & =\left(\lambda -a_{11}\right)\left[\left(\lambda -a_{22}\right)\left(\lambda -a_{33}\right)-a_{23}a_{32}\right]-a_{12}a_{21}\left(\lambda -a_{33}\right)\\ Q(\lambda ) & =-\left(\lambda -a_{11}\right)\left[\left(\lambda -a_{22}\right)b_{33}+a_{32}b_{23}\right]+a_{12}a_{21}b_{33} \end{array}$$

$d_{2}$ is modified due to $x_{4}^{*}=0$ and $x_{6}^{*}=0$. The terms $a_{44},a_{46},a_{64},a_{66},a_{86}$ are affected. Specifically, $a_{46}$ becomes 0 because it depends on $x_{2}^{*}$, but the term $x_{6}^{*}$ is 0 in $E_{3}$. Also, $a_{42}$ is 0. Therefore, $d_{2}$ is

(28) $$d_{2}=\left|\begin{array}{ccccc} \lambda -a_{44} & 0 & 0 & 0 & 0\\ 0 & \lambda -a_{55} & 0 & -a_{57} & 0\\ -a_{64} & 0 & \lambda -a_{66} & 0 & 0\\ 0 & -a_{75} & 0 & \lambda -a_{77} & 0\\ 0 & 0 & -a_{86} & -a_{87} & \lambda -a_{88} \end{array}\right|=0$$

Also, at $E_{3}$, we have $a_{44}=-\gamma_{1}$ and $a_{66}=-\mu_{1}$. The terms $a_{46}$ and $a_{86}$ are zero. The determinant $d_{2}$ simplifies significantly:

$$\begin{array}{cc} d_{2} & =\left(\lambda -a_{44}\right)\left|\begin{array}{cccc} \lambda -a_{55} & 0 & -a_{57} & 0\\ 0 & \lambda -a_{66} & 0 & 0\\ -a_{75} & 0 & \lambda -a_{77} & 0\\ 0 & -a_{86} & -a_{87} & \lambda -a_{88} \end{array}\right|\\ \; & =\left(\lambda +\gamma_{1}\right)\left(\lambda +\mu_{1}\right)\left|\begin{array}{ccc} \lambda -a_{55} & -a_{57} & 0\\ -a_{75} & \lambda -a_{77} & 0\\ 0 & -a_{87} & \lambda -a_{88} \end{array}\right| \end{array}$$

This further simplifies to

$$d_{2}=\left(\lambda +\gamma_{1}\right)\left(\lambda +\mu_{1}\right)\left(\lambda -a_{88}\right)\left|\begin{array}{cc} \lambda -a_{55} & -a_{57}\\ -a_{75} & \lambda -a_{77} \end{array}\right|=0$$

Thus, the eigenvalues from this part are $-\gamma_{1}$, $-\mu_{1}$, −ω (since $a_{88}=-\omega$), and the roots of the 2×2 determinant:

$$\left(\lambda -a_{55}\right)\left(\lambda -a_{77}\right)-a_{57}a_{75}=0$$

In this case, $d_{3}$ as defined for $E_{2}$ does not appear in the same form. Instead, the part of the characteristic equation related to the rumor dynamics ($x_{4},x_{5},x_{6},x_{7},x_{8}$) decouples into simpler terms. The stability of $E_{3}$ depends on the roots of $d_{1}=0$ and the roots of the simplified $d_{2}=0$.

When τ=0, the stability analysis for $E_{3}$ and $E_{4}$ is similar to the stability analysis carried out for $E_{2}$.

### 4.5. Characteristic Equation for $E_{4}$

For the equilibrium point $E_{4}$, we have $x_{5}^{*}=0$ and $x_{7}^{*}=0$. This implies that the terms $a_{52}$ and $a_{57}$ (which depend on $x_{7}^{*}$) become zero. Similarly, $a_{75}$ and $a_{87}$ are also affected. The Jacobian matrix simplifies, and the characteristic equation can be analyzed by considering the decoupled parts.

The characteristic equation still factors into $d_{1}=0$ and a modified $d_{2}=0$. The $d_{1}$ part remains the same as for $E_{2}$ and $E_{3}$:

(29) $$d_{1}=\left|\begin{array}{ccc} \lambda -a_{11} & -a_{12} & 0\\ -a_{21} & \lambda -a_{22} & -b_{23}e^{-\lambda \tau }\\ 0 & -a_{32} & \lambda -a_{33}-b_{33}e^{-\lambda \tau } \end{array}\right|=0$$

Again, the coefficients $a_{ij}$ and $b_{ij}$ are evaluated at $E_{4}$.

The $d_{2}$ part of the characteristic equation is modified due to $x_{5}^{*}=0$ and $x_{7}^{*}=0$. The terms $a_{55},a_{57},a_{75},a_{77},a_{87}$ are affected. Specifically, $a_{57}$ becomes 0. Also, $a_{52}$ is 0. The $d_{2}$ determinant simplifies to the following:

(30) $$d_{2}=\left|\begin{array}{ccccc} \lambda -a_{44} & 0 & -a_{46} & 0 & 0\\ 0 & \lambda -a_{55} & 0 & 0 & 0\\ -a_{64} & 0 & \lambda -a_{66} & 0 & 0\\ 0 & 0 & 0 & \lambda -a_{77} & 0\\ 0 & 0 & -a_{86} & 0 & \lambda -a_{88} \end{array}\right|=0$$

Given $x_{5}^{*}=0$ and $x_{7}^{*}=0$ at $E_{4}$, we have $a_{55}=-\gamma_{2}$ and $a_{77}=-\mu_{2}$. The terms $a_{57}$ and $a_{87}$ are zero. The determinant $d_{2}$ simplifies significantly:

$$\begin{array}{cc} d_{2} & =\left(\lambda -a_{55}\right)\left|\begin{array}{cccc} \lambda -a_{44} & -a_{46} & 0 & 0\\ -a_{64} & \lambda -a_{66} & 0 & 0\\ 0 & 0 & \lambda -a_{77} & 0\\ 0 & -a_{86} & 0 & \lambda -a_{88} \end{array}\right|\\ \; & =\left(\lambda +\gamma_{2}\right)\left(\lambda +\mu_{2}\right)\left|\begin{array}{ccc} \lambda -a_{44} & -a_{46} & 0\\ -a_{64} & \lambda -a_{66} & 0\\ -a_{86} & 0 & \lambda -a_{88} \end{array}\right| \end{array}$$

This further simplifies to

$$d_{2}=\left(\lambda +\gamma_{2}\right)\left(\lambda +\mu_{2}\right)\left(\lambda -a_{88}\right)\left|\begin{array}{cc} \lambda -a_{44} & -a_{46}\\ -a_{64} & \lambda -a_{66} \end{array}\right|=0$$

Thus, the eigenvalues from this part are $-\gamma_{2}$, $-\mu_{2}$, −ω, and the roots of the 2×2 determinant are as follows:

$$\left(\lambda -a_{44}\right)\left(\lambda -a_{66}\right)-a_{46}a_{64}=0$$

Similar to $E_{3}$, the term $d_{3}$ as defined for $E_{2}$ does not appear in the same form for $E_{4}$. The part of the characteristic equation related to the rumor dynamics ($x_{4},x_{5},x_{6},x_{7},x_{8}$) decouples into simpler terms.

### 4.6. Characteristic Equation for $E_{5}$

For the equilibrium point $E_{5}$, we have $x_{4}^{*}=x_{5}^{*}=x_{6}^{*}=x_{7}^{*}=x_{8}^{*}=0$. This significantly simplifies the Jacobian matrix. In this case, the terms $a_{46}$ and $a_{57}$ become zero. The Jacobian matrix at $E_{5}$ can be permuted to a block diagonal form, where the dynamics of $x_{1},x_{2},x_{3}$ are decoupled from the dynamics of $x_{4},x_{5},x_{6},x_{7},x_{8}$.

The characteristic equation for $E_{5}$ can be written as the product of the characteristic equations of these decoupled blocks. The eigenvalues corresponding to the rumor dynamics ($x_{4},x_{5},x_{6},x_{7},x_{8}$) are simply the diagonal entries of the Jacobian submatrix for these variables, which are $-\gamma_{1},-\gamma_{2},-\mu_{1},-\mu_{2},-\omega$. All these eigenvalues are negative, implying that the rumor-free state is stable with respect to the rumor variables.

Therefore, the stability of $E_{5}$ is determined only by the eigenvalues of the 3×3 submatrix corresponding to $x_{1},x_{2},x_{3}$. The characteristic equation for $E_{5}$ is as follows:

(31) $$\left|\begin{array}{ccc} \lambda -a_{11} & -a_{12} & 0\\ -a_{21} & \lambda -a_{22} & -b_{23}e^{-\lambda \tau }\\ 0 & -a_{32} & \lambda -a_{33}-b_{33}e^{-\lambda \tau } \end{array}\right|=0$$

While the general form of $d_{3}$ using the Schur complement method is used for $E_{2},$ where all components are nonzero and coupled, for $E_{5}$, the terms that constitute $d_{3}$ become zero.

### 4.7. Stability Analysis for $E_{3},E_{4},E_{5}$ When τ>0

For $E_{3},E_{4}$, the characteristic equation is given by $d_{1}=P\left(\lambda \right)+Q\left(\lambda \right)e^{-\lambda \tau }=0$, where P(λ) and Q(λ) are evaluated at the specific coordinates of $E_{3},E_{4}$, respectively. The methodology for analyzing stability when τ>0 is identical to that used for $E_{2}$.

For $E_{5}$, P(λ) and Q(λ) are derived from the 3×3 submatrix for $x_{1},x_{2},x_{3}$ evaluated at $E_{5}$. The methodology for analyzing stability for τ>0 is identical to that for $E_{2}$, $E_{3}$, and $E_{4}$.

## 5. Numerical Analysis

Table 1 shows the main parameters used in the numerical analysis, together with their values and descriptions. Some values were taken from the literature [2], while others were estimated to give realistic results that match the behavior expected from dynamical systems theory.

Using MATLAB (R2024b; MathWorks, Natick, MA, USA), and the parameters from Table 1, the numerical values of partial derivatives are

$$a_{11}=-0.141507,\;a_{22}=0,\;a_{21}=0.0715074,\;a_{23}=0,\;a_{32}=1.87336,$$

$$a_{33}=-0.07,\;b_{23}=-0.0429045,\;b_{33}=0.0429045,\;a_{12}=-1.94336$$

**$E_{1}$ (Rumor-free equilibrium):**

$$E_{1}=\left(7.857142857,0,0,0,0,0,0,0\right)$$

**$E_{2}$ (Endemic equilibrium):**

$$E_{2}=\left(3.886721810,0.143014850,6.244536360,0.241713020,0.241713020,8.459955590,8.459955590,0.845995559\right)$$

**$E_{3}$ (Rumor 1 dies; Rumor 2 persists):**

$$E_{3}=\left(3.635051610,0.162609180,5.825086010,0,0.353120790,0,12.359227600,0.617961380\right)$$

**$E_{4}$ (Rumor 2 dies; Rumor 1 persists):**

$$E_{4}=\left(3.635051610,0.162609180,5.825086010,0.353120790,0,12.359227600,0,0.617961380\right)$$

**$E_{5}$ (Both rumors die out, but individuals remain active):**

$$E_{5}=\left(2.946428,0.233333,4.67738,0,0,0,0,0\right)$$

### 5.1. Stability Condition of $E_{1}$

We check the condition $\mu^{2}>\alpha \Lambda$:

$$\mu^{2}={\left(0.07\right)}^{2}=0.0049$$

αΛ=0.5×0.55=0.275

Since 0.0049<0.275, we have $\mu^{2}<\alpha \Lambda$; therefore, $E_{1}$ is unstable.

### 5.2. Routh-Hurwitz Analysis for τ=0 at $E_{2}$

We present the Routh–Hurwitz stability results for the endemic equilibrium point $E_{2}$ at τ=0, based on the rank-one perturbation method applied to the corresponding subsystem. The coefficients were computed in MATLAB using the parameter values listed in Table 1.

- $A_{1}=0.168603$;
- $A_{2}=0.223174$;
- $A_{3}=0.015139$.

Routh–Hurwitz stability conditions:

1. $A_{1}=$0.168603>0 satisfied.
2. $A_{3}=$0.015139>0, satisfied.
3. $A_{1}A_{2}-A_{3}=0.0224888>0.$ This condition is also satisfied.

We observe that this analysis, based on the rank-one perturbation method, aligns with the numerical simulations performed in the absence of delays. All three Routh–Hurwitz stability conditions are met for the given numerical coefficients. This confirms that, in the absence of time delay (τ=0), the endemic equilibrium $E_{2}$ is locally asymptotically stable.

- Using a Python script (version 3.11; Python Software Foundation, Wilmington, DE, USA) to solve Equation (26) for $r_{c}$ and subsequently calculate $\tau_{critic}$, we obtained the following results:
  $r_{c}\approx 0.43509283147507116$;
  $\cos(r_{c}\tau )\approx 0.7970167196266242$;
  $\sin(r_{c}\tau )\approx 0.6039572407344895$;
  $\tau_{critic}\approx 1.4903874368417422$.

We evaluate the complex values of $P(ir_{c})$, $Q(ir_{c})$, $P^{\prime }\left(ir_{c}\right)$, and $Q^{\prime }\left(ir_{c}\right)$ using the provided formulas and parameters.

Our values are as follows:

- $P(ir_{c})\approx -0.03031197-0.01759327i$;
- $Q(ir_{c})\approx 0.01353357+0.03232926i$;
- $P^{\prime }\left(ir_{c}\right)\approx -0.41904721+0.18405036i$;
- $Q^{\prime }\left(ir_{c}\right)\approx 0.07430429-0.03733488i$.

We compute the derivative $\frac{d\lambda }{d\tau }$ at the critical point:

$$\frac{d\lambda }{d\tau }{|}_{\lambda =ir_{c}}\approx 0.00055760+0.00042001i$$

Our interest is the real part of this derivative, which determines the direction of crossing of the imaginary axis:

Redλdτλ=irc≈0.00055760

### 5.3. Existence Condition for $x_{3}^{*}>0$ at $E_{5}$

αΛη=0.5×0.55×0.3=0.0825

$\mu^{2}\left(\alpha +\eta \right)={\left(0.07\right)}^{2}\times \left(0.5+0.3\right)=0.0049\times 0.8=0.00392$

Since 0.0825>0.00392, the condition $\alpha \Lambda \eta >\mu^{2}\left(\alpha +\eta \right)$ is satisfied, confirming the positivity of $x_{3}^{*}$ for $E_{5}$ with these parameters.

## 6. MATLAB Simulations

**Figure 1:**
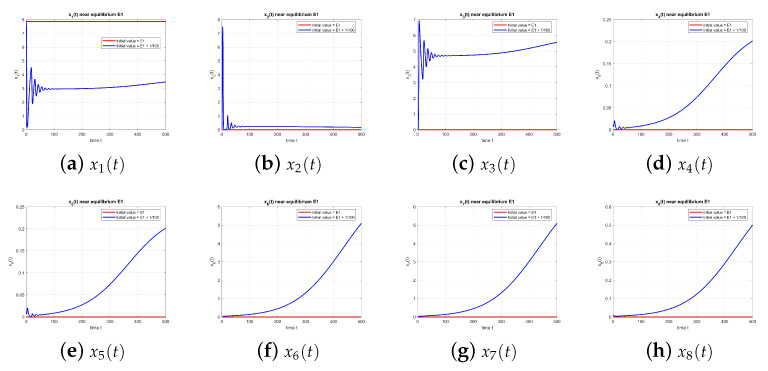
Equilibrium point $E_{1}$ at τ=0 with and without perturbation. $E_{1}$ is unstable.

Figure 1 shows the behavior of the equilibrium point $E_{1}$ at τ=0, with and without perturbation. One can see that $E_{1}$ is unstable.

**Figure 2:**
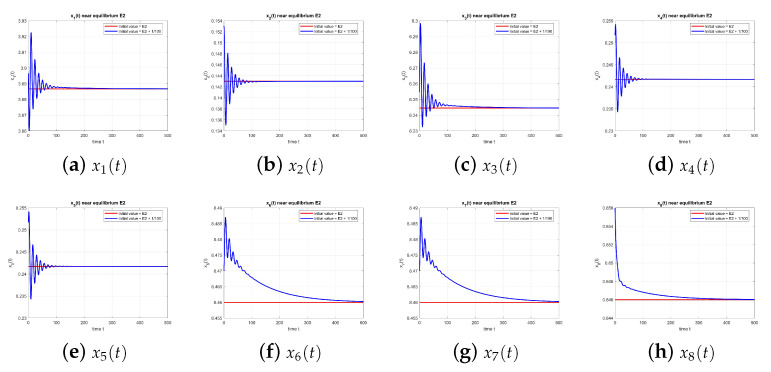
Equilibrium point $E_{2}$ at τ=0, with and without perturbation. $E_{2}$ is stable. *Note:* with τ=0, there is no delay-induced mechanism; accordingly, no delay-induced sustained oscillations appear, and trajectories converge to the equilibrium.

As shown in Figure 2, the dynamics of the equilibrium point $E_{2}$ at τ=0 converge to the equilibrium, confirming stability.

**Figure 3:**
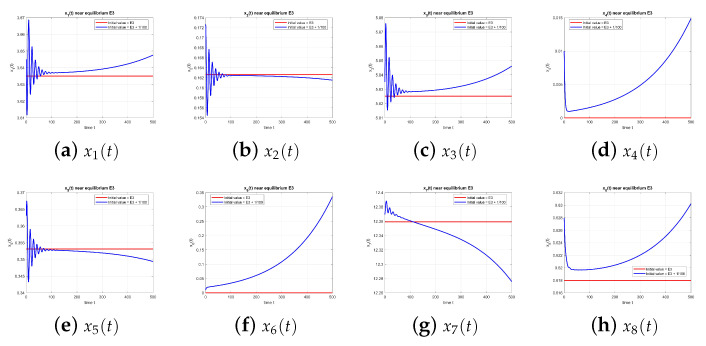
Equilibrium point $E_{3}$ at τ=0, with and without perturbation. $E_{3}$ is unstable. *Note:* without delay (τ=0), no sustained oscillations occur, and trajectories diverge from the equilibrium.

As illustrated in Figure 3, the equilibrium point $E_{3}$ is unstable at τ=0.

**Figure 4:**
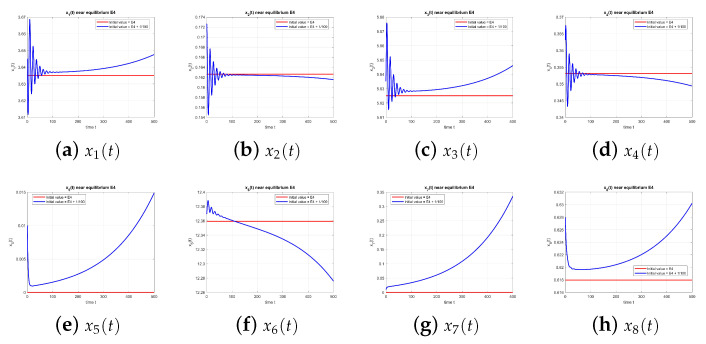
Equilibrium point $E_{4}$ at τ=0, with and without perturbation. $E_{4}$ is unstable. *Note:* with τ=0, there is no delay-induced mechanism; accordingly, no delay-induced sustained oscillations appear, and trajectories depart from the equilibrium according to the local eigenstructure.

Figure 4 shows that $E_{4}$ is also unstable when τ=0.

**Figure 5:**
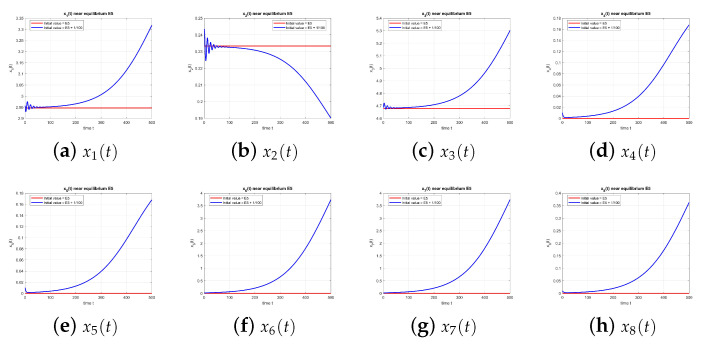
Equilibrium point $E_{5}$ at τ=0, with and without perturbation. $E_{5}$ is unstable. *Note:* with τ=0, there is no delay-induced mechanism; accordingly, no delay-induced sustained oscillations appear. The dynamics are governed by the instantaneous $\left(x_{1},x_{2},x_{3}\right)$ subsystem, and trajectories depart from the equilibrium according to its local eigenstructure.

Figure 5 shows that $E_{5}$ is unstable when τ=0.

**Figure 6:**
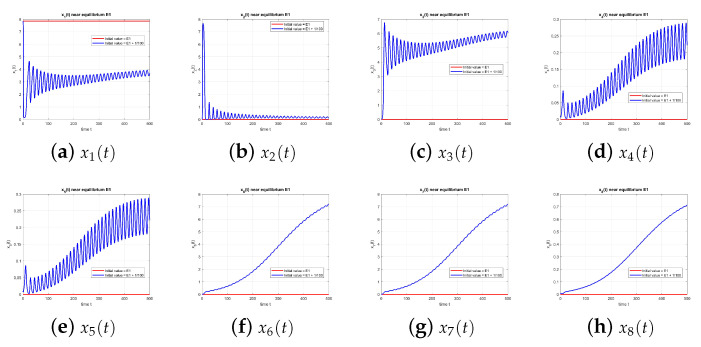
Equilibrium point $E_{1}$ at τ=1.49 with and without perturbation. $E_{1}$ is unstable.

Figure 6 shows the dynamics of equilibrium $E_{1}$ at τ=1.49, where instability arises.

**Figure 7:**
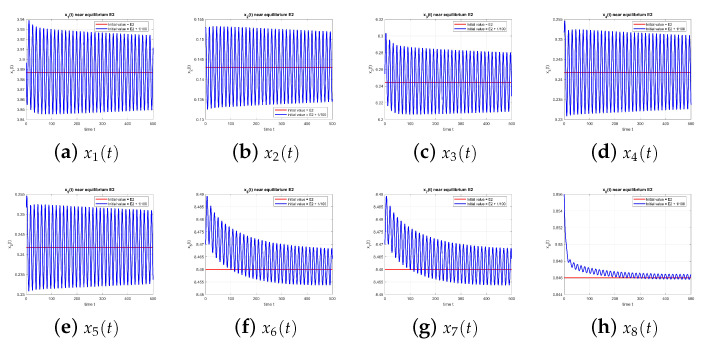
Equilibrium point $E_{2}$ at τ=1.49, with and without perturbation. $E_{2}$ is unstable. *Mechanism:* a delay-induced negative feedback in the $x_{2}$–$x_{3}$ loop generates a Hopf bifurcation; the two rumor components introduce a second oscillatory mode whose weak coupling to the first modulates amplitudes.

As illustrated in Figure 7, equilibrium $E_{2}$ becomes unstable at τ=1.49 due to delay effects.

**Figure 8:**
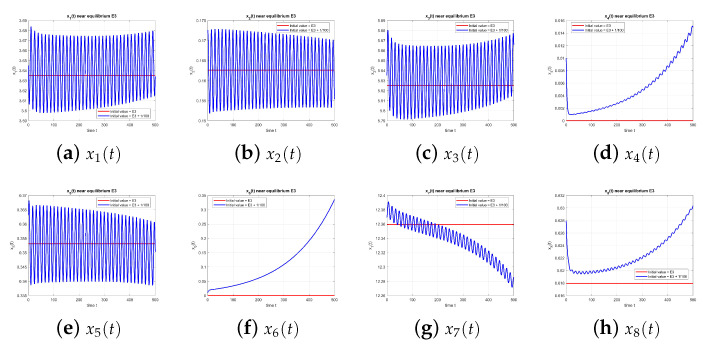
Equilibrium point $E_{3}$ at τ=1.49, with and without perturbation. $E_{3}$ is unstable. *Mechanism:* a delay-induced negative feedback in the $x_{2}$–$x_{3}$ loop generates a Hopf bifurcation. Since Rumor 1 is absent ($x_{4}=x_{6}=0$), oscillations are driven primarily by the disengagement delay, with no cross-rumor modulation.

Figure 8 shows instability of equilibrium $E_{3}$ at τ=1.49.

**Figure 9:**
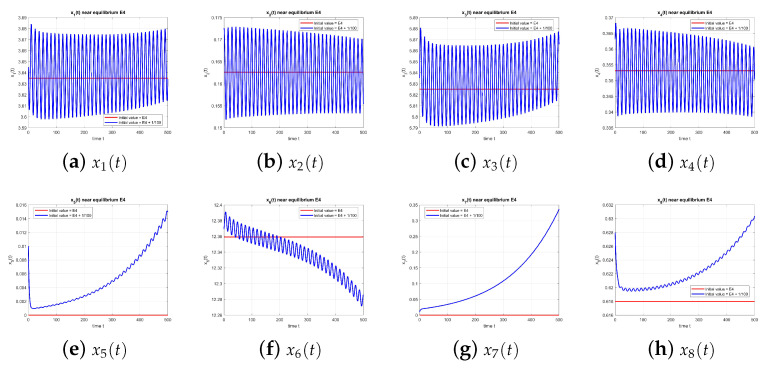
Equilibrium point $E_{4}$ at τ=1.49, with and without perturbation. $E_{4}$ is unstable. *Mechanism:* a delay-induced negative feedback in the $x_{2}$–$x_{3}$ loop generates a Hopf bifurcation. Since Rumor 2 is absent ($x_{5}=x_{7}=0$), oscillations are driven by the disengagement delay, with no cross-rumor modulation.

As indicated in Figure 9, equilibrium $E_{4}$ is unstable at τ=1.49.

**Figure 10:**
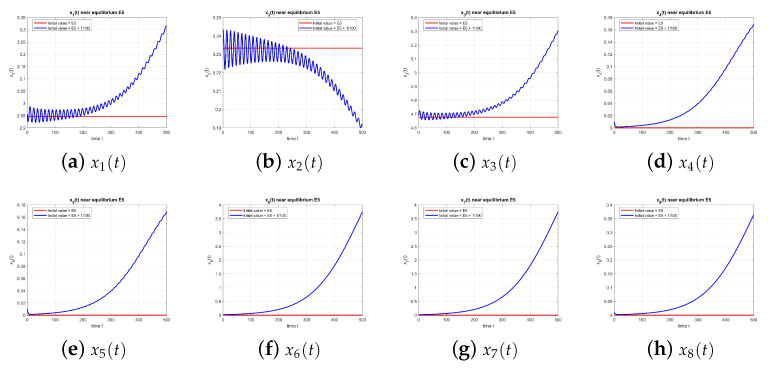
Equilibrium point $E_{5}$ at τ=1.49, with and without perturbation. $E_{5}$ is unstable. *Mechanism:* oscillations are generated exclusively by the delayed negative feedback in the $x_{2}$–$x_{3}$ loop; no rumor components are active ($x_{4}=x_{5}=x_{6}=x_{7}=x_{8}=0$), so there is no cross-rumor mode.

Figure 10 illustrates instability of equilibrium $E_{5}$ at τ=1.49.

**Figure 11:**
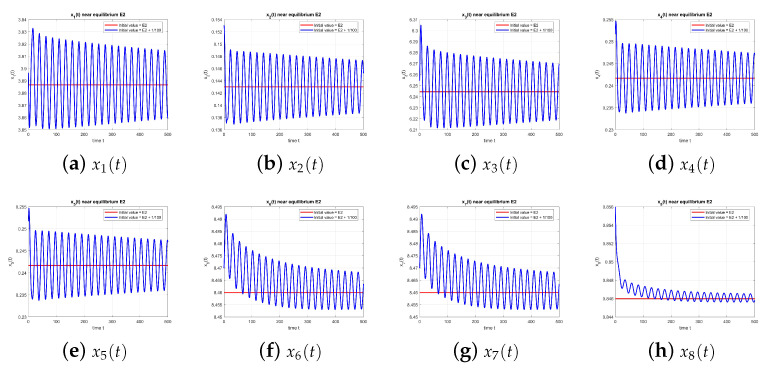
Equilibrium point $E_{2}$ at τ=9.39, with and without perturbation. $E_{2}$ is stable. *Mechanism:* the same delayed negative feedback in the $x_{2}$–$x_{3}$ loop induces transient oscillations; for this τ, the equilibrium is stable, and the oscillations decay. The two rumor components add a weakly coupled mode that can modulate amplitudes.

As shown in Figure 11, equilibrium $E_{2}$ is stable at τ=9.39.

**Figure 12:**
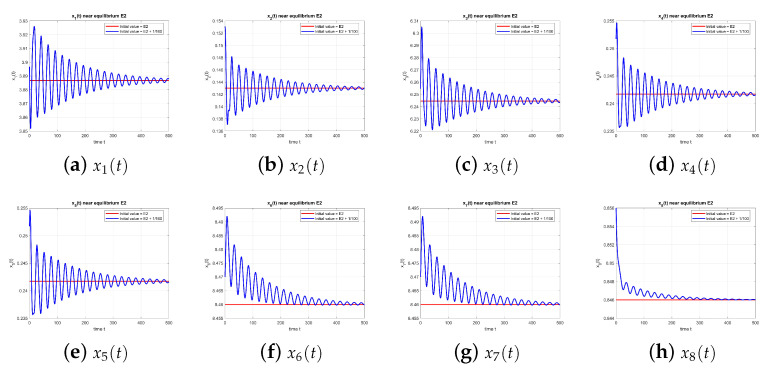
Equilibrium point $E_{2}$ at τ=10.5, with and without perturbation. $E_{2}$ is stable again. *Mechanism:* the same delayed negative feedback in the $x_{2}$–$x_{3}$ loop induces transient oscillations; for this τ, the equilibrium is stable, and the oscillations decay. The two rumor components add a weakly coupled mode that can modulate amplitudes.

As shown in Figure 12, the equilibrium point $E_{2}$ at τ=10.5 is stable again, with and without perturbation.

**Figure 13:**
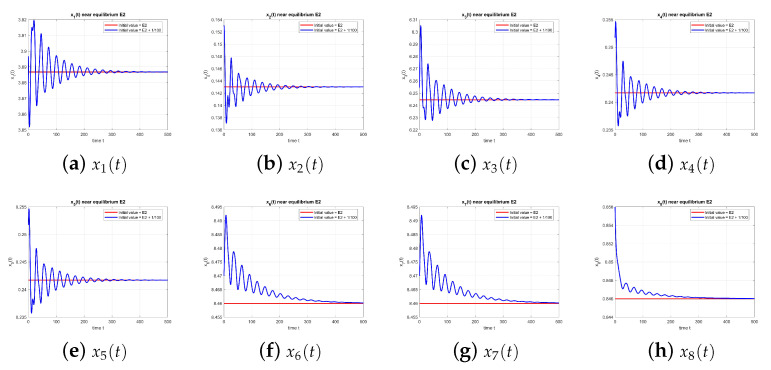
Equilibrium point $E_{2}$ at τ=12.0, with and without perturbation. $E_{2}$ remains stable. *Mechanism:* the same delayed negative feedback in the $x_{2}$–$x_{3}$ loop induces transient oscillations; for this τ, the equilibrium is stable, and the oscillations decay. The two rumor components add a weakly coupled mode that can modulate amplitudes.

As shown in Figure 13, the equilibrium point $E_{2}$ at τ=12.0 remains stable, with and without perturbation.

### 6.1. Discussion of Oscillatory Dynamics and Feedback Loops

The oscillations originate in a delayed negative feedback in the $x_{2}$–$x_{3}$ subsystem: increases in active users $x_{2}$ raise the disconnected class $x_{3}$ after a lag τ, which suppresses $x_{2}$ and rumor incidence; the subsequent relaxation allows $x_{2}$ to rebuild. This generates a Hopf bifurcation at τ≈1.49 (purely imaginary root with dℜλ/dτ>0). The coexistence of two rumor components introduces a second oscillatory mode weakly coupled through shared susceptibles and the skepticism pool, producing amplitude modulation and quasi-periodic-like transients in some parameter ranges.

### 6.2. Academic Implications

(i) A compact two-rumor DDE with saturating incidence yields a single quadratic in $x_{2}^{*}$ that governs endemicity and compartment positivity.

(ii) A rank-one perturbation splits the 8D characteristic equation into a 3×3 delayed core and a rumor block, enabling Routh–Hurwitz tests at τ=0 and geometric stability-switch criteria for τ>0.

(iii) We locate the first Hopf threshold and show multiple stability windows as τ varies, explaining the delayed negative-feedback mechanism that drives oscillations and amplitude modulation.

### 6.3. Practical Implications

From the model, there are a few simple actions that can help stop a rumor from growing:

1. **React quickly:** The faster false or doubtful posts are checked and answered, the less chance the rumor has to spread (in the model, smaller delay τ.)
2. **Make sharing a bit slower:** Small limits, like short waits before re-sharing or a few extra clicks, can reduce how fast the rumor reaches new people (in the model, lower spreading rate, smaller peaks in $x_{2}$.)
3. **Encourage doubt:** Show clear corrections, ask users to check the source, and give an easy way to stop following the topic. This helps people leave the “believer” group (in the model, higher $\mu_{1},\mu_{2},\omega$.)
4. **Step in during busy moments:** If activity on a rumor suddenly jumps, reduce its visibility for a short time. This can stop the quick rise and fall cycles (in the model, keeping the $x_{2}$–$x_{3}$ loop stable.)
5. **Check if a rumor can survive:** A rumor will fade if
  $$\beta_{i}\;x_{2}^{*}\;<\;\mu_{i}\left(1+\alpha_{i}x_{2}^{*}\right).$$
  This means the rate of spreading is lower than the rate of people leaving or doubting it. You can reach this by lowering $\beta_{i}$ or $x_{2}^{*}$, or by raising $\mu_{i}$ and ω.

## 7. Conclusions

In this study, we analyzed a mathematical model of rumor spread on social media, highlighting the impact of delays, rumor incidence rates, and user skepticism on rumor propagation dynamics. Our results, supported by numerical simulations and stability analyses, align with similar research, confirming and expanding previous insights.

We developed and analyzed a delay differential model to explore the dynamics of two coexisting rumors spreading through an online social media (OSM) platform.

The existence and stability of the rumor present equilibrium ($E_{2}$) depends on certain critical boundary conditions that control rumor persistence, similar to the basic reproduction number in epidemic models. This is just like how diseases spread if the infection rate crosses a certain limit. In simple terms, our model shows that if enough people start sharing a rumor, it will not just disappear; instead, it will keep spreading continuously throughout the network [20].

Our research shows that time delays play an important role in how rumors spread. Similar to previous research [8], we found that delays, such as hesitation periods or time before users respond to misinformation, can create complex behaviors, including oscillations or periodic rumor outbreaks (Hopf bifurcations), which make it harder to stop rumors once they appear.

We also found that adding skepticism into our model is similar to vaccination effects in disease models, helping reduce how widely rumors spread. When a network has enough informed or skeptical users who question suspicious content, false information struggles to spread. This reinforces the need for educating the public and providing timely responses to stop rumors before they take hold.

Real-world studies on social media strongly support our results. For example, research by Vosoughi [21] showed that false information spreads faster and deeper than verified information. This happens because of how people react and share content, not due to automated systems.

Looking at how networks are built gives us useful insights. In networks where people are highly connected, rumors spread fast. But if there are enough skeptical people, they can slow things down. This suggests that focusing efforts on key areas of a network can be an effective way to stop misinformation.

However, these solutions have challenges because of how people think. For example, people tend to believe information that matches their opinions, and sometimes, correcting them makes them hold onto false ideas even more. This means that fighting misinformation needs a flexible approach, constant improvements, and strategies based on real evidence to avoid unexpected problems.

- Our analysis identifies multiple equilibria: the rumor-free equilibrium ($E_{1}$); the endemic equilibrium ($E_{2}$), where both rumors persist; single-rumor equilibria ($E_{3}$ and $E_{4}$), where only one rumor survives; and the equilibrium $E_{5}$, where both rumors die out, but individuals remain active.
- The existence of $E_{2}$ depends on a key condition related to the population of disconnected users ($x_{3}^{*}$):
  $$x_{3}^{*}=\frac{\alpha x_{1}^{*}-\mu }{\eta }>0\;\Rightarrow \;\alpha x_{1}^{*}>\mu .$$
- This condition means that new users need to start spreading the rumor ($\alpha x_{1}^{*}$) faster than they leave the system naturally (μ). If this inequality does not hold, $E_{2}$ will not exist, meaning that the rumor dies out, and the system returns to the rumor-free state.
- Stability analysis, via the characteristic equation $\det(\lambda I-\frac{\partial f}{\partial x}-\frac{\partial f}{\partial x_{\tau }}e^{-\lambda \tau })=0$ and guided by the Routh–Hurwitz criterion, helps us understand how the system behaves over time. For $E_{1}$, stability requires $\alpha \frac{\Lambda }{\mu }-\mu <0$, which translates to $\alpha \Lambda <\mu^{2}$.
- However, numerical evaluation indicates the instability of $E_{1}$ since $\alpha \Lambda =0.275>\mu^{2}=0.0049$, revealing that even a small number of rumor adopters pushes the system away from the rumor-free state.
- The numerical results for $E_{2}$ present an interesting and complex scenario:
- If the delay τ becomes too high, $E_{2}$ can lose stability, leading the system to oscillate away from the endemic state. The critical value $\tau_{critic}\approx 1.4903874368417422$, computed by solving the characteristic equation, indicates the existence of a critical period before the system becomes unstable.
- The numerical calculation shows that the real part of $\frac{d\lambda }{d\tau }$ at the critical point is approximately 0.00055760, which is a positive value. This positive sign indicates that as the delay τ increases beyond its critical value ($\tau_{critic}$), the real part of the dominant eigenvalue increases, causing the system to lose stability.
- Based on the numerical simulations for $E_{2}$ at τ=1.49, τ=9.39, τ=10.5, and τ=12, the observed behavior strongly indicates a sequence of Hopf bifurcations rather than a single forward or backward bifurcation. The system exhibits a complex dynamic where stability is lost and then regained as the delay parameter τ increases.
- **Initial stability (τ<1.49):** For small values of τ (e.g., τ=0), the equilibrium $E_{2}$ is locally asymptotically stable.
- **First Hopf bifurcation (τ=1.49):** At τ=1.49, the simulations clearly show that $E_{2}$ loses its stability, indicating a **forward Hopf bifurcation.**
- **Re-stabilization and later Hopf bifurcations (τ=9.39 and τ=10.5):** This means that the system became stable again after being unstable. The change happens when some eigenvalues move back to the stable side. This can happen in different ways:
  1. **Another supercritical Hopf bifurcation:** It is possible that at τ=9.39, another pair of eigenvalues crosses the imaginary axis, leading to a new supercritical Hopf bifurcation where the equilibrium regains stability, and the previously existing stable limit cycle disappears or becomes unstable. It is less common for the same equilibrium to regain stability via a supercritical bifurcation unless there are multiple pairs of eigenvalues involved.
  2. **Subcritical (backward) Hopf bifurcation:** As the delay τ increases, an unstable loop (limit cycle) can meet the equilibrium and vanish; then, the equilibrium is stable again.
  3. **Multiple stability switches:** In DDEs, it is common to observe multiple stability switches as the delay parameter increases. Each switch corresponds to a pair of eigenvalues crossing the imaginary axis. The re-stabilization at τ=9.39 and τ=10.5 suggests that the system undergoes further Hopf bifurcations, where the stability of $E_{2}$ changes again. The exact nature (supercritical or subcritical) of these subsequent bifurcations would require a more detailed mathematical analysis, such as computing the first Lyapunov coefficient at each critical point.
- For DDEs, the calculation of the first Lyapunov coefficient is more involved than for ordinary differential equations (ODEs) due to the infinite-dimensional nature of the phase space. It typically requires the use of the center manifold theory and the normal form theory for DDEs. The general formula for the first Lyapunov coefficient at a Hopf bifurcation point for DDEs can be found in specialized research, such as the works by Hassard et al. [22].

## Figures and Tables

**Table 1:** Model parameters for rumor spreading.

Parameter	Value	Description
Λ	0.55	Birth rate into $x_{1}$ (potential users)
α	0.5	Rate at which potential users $x_{1}$ become active users $x_{2}$, given contact with $x_{2}$
μ	0.07	Natural death or leaving rate [2]
η	0.3	Rate at which active users $x_{2}$ and non-users $x_{3}$ interact, causing movement between them
$\beta_{1}$	0.1	Parameter controlling how Rumor 1 spreads
$\alpha_{1}$	0.05	Parameter controlling how Rumor 1 spreads
$\epsilon_{1}$	0.05	Parameter controlling how Rumor 1 spreads
$\beta_{2}$	0.1	Parameter controlling how Rumor 2 spreads
$\alpha_{2}$	0.05	Parameter controlling how Rumor 2 spreads
$\epsilon_{2}$	0.05	Parameter controlling how Rumor 2 spreads
$\gamma_{1}$	0.35	Progression rate from $x_{4}$ to $x_{6}$ [2]
$\gamma_{2}$	0.35	Progression rate from $x_{5}$ to $x_{7}$ [2]
$\mu_{1}$	0.01	Rate at which believers ($x_{6}$) become skeptics ($x_{8}$)
$\mu_{2}$	0.01	Rate at which believers ($x_{7}$) become skeptics ($x_{8}$)
ω	0.2	Rate at which skeptics ($x_{8}$) leave skepticism and either stop using the platform or disconnect ($x_{3}$)

## Data Availability

All data supporting the findings of this study are contained within the article. In addition, the MATLAB codes used for the numerical simulations are available from the corresponding author upon reasonable request.
